# Implementation of a comprehensive safer conception intervention for HIV‐serodiscordant couples in Kenya: uptake, use and effectiveness

**DOI:** 10.1002/jia2.25261

**Published:** 2019-04-07

**Authors:** Renee Heffron, Kenneth Ngure, Jennifer Velloza, Catherine Kiptinness, Justice Quame‐Amalgo, Lynda Oluch, Nicholas Thuo, John Njoroge, Richard Momanyi, Stephen Gakuo, Sarah Mbugua, Susan Morrison, Harald Haugen, Bhavna Chohan, Connie Celum, Jared M Baeten, Nelly Mugo

**Affiliations:** ^1^ Department of Global Health University of Washington Seattle WA USA; ^2^ Department of Epidemiology University of Washington Seattle WA USA; ^3^ Jomo Kenyatta University of Agriculture and Technology Nairobi Kenya; ^4^ Partners in Health and Research Development Centre for Clinical Research Kenya Medical Research Institute Nairobi Kenya; ^5^ Department of Medicine University of Washington Seattle WA USA; ^6^ Center for Clinical Research Kenya Medical Research Institute Nairobi Kenya

**Keywords:** HIV‐serodiscordant couples, safer conception, pregnancy, PrEP, ART, fertility

## Abstract

**Introduction:**

Safer conception strategies minimize HIV risk during condomless sex to become pregnant. Gaps remain in understanding the acceptability, feasibility and choices HIV‐serodiscordant couples make when multiple safer conception options are available.

**Methods:**

We conducted a pilot study of a comprehensive safer conception package for HIV‐serodiscordant couples with immediate fertility desires in Kenya from March 2016 to April 2018. The intervention package included antiretroviral therapy (ART) for HIV‐positive partners, oral pre‐exposure prophylaxis (PrEP) for HIV‐negative partners, daily fertility and sexual behaviour tracking via short message service (SMS) surveys, counselling on self‐insemination, and referrals for voluntary medical male circumcision and fertility care. Couples attended monthly visits until pregnancy with HIV testing for negative partners at each visit. We estimated the number of expected HIV seroconversions using a counterfactual cohort simulated from gender‐matched couples in the placebo arm of a previous PrEP clinical trial. We used bootstrap methods to compare expected and observed seroconversions.

**Results:**

Of the 74 enrolled couples, 54% were HIV‐negative female/HIV‐positive male couples. The 6 and 12‐month cumulative pregnancy rates were 45.3% and 61.9% respectively. In the month preceding pregnancy, 80.9% of HIV‐positive partners were virally suppressed, 81.4% of HIV‐negative partners were highly adherent to PrEP, and SMS surveys indicated potential timing of condomless sex to peak fertility (median of sex acts = 10, interquartile range (IQR) 7 to 12; median condomless sex acts = 3.5, IQR 1 to 7). Most (95.7%) pregnancies were protected by ≥2 strategies: 57.4% were protected by high PrEP and ART adherence, male circumcision with or without timed condomless sex; 10 (21.3%) were protected by viral suppression in the HIV‐positive partner and male circumcision with or without timed condomless sex; 8 (17.0%) were protected by high PrEP adherence and male circumcision with or without timed condomless sex. We observed 0 HIV seroconversions (95% CI 0.0 to 6.0 per 100 person years), indicating a 100% reduction in HIV risk (*p* = 0.04).

**Conclusions:**

The use of multiple safer conception strategies, primarily PrEP, ART, male circumcision and/or tracking fertility, was acceptable and feasible for African HIV‐serodiscordant couples and significantly reduced HIV transmission risk. It is important to increase the availability of and counselling about safer conception services in regions with HIV epidemics involving heterosexual transmission and high fertility.

## 1 Introduction

Attempting pregnancy is a reproductive right and a critical goal for many couples 1, 2. For heterosexual couples with one partner living with HIV, pregnancy attempts are often through condomless sex, which could facilitate sexual HIV transmission. Multiple strategies are available to reduce HIV transmission risk during pregnancy attempts via condomless sex and complementary strategies are available to optimize fertility 3. These “safer conception” strategies include: the use of antiretroviral therapy (ART) by the partner living with HIV 4, pre‐exposure prophylaxis (PrEP) use by the HIV‐negative partner 5, diagnosis and appropriate treatment of sexually transmitted infections (STI) 6, voluntary medical male circumcision (VMMC) 7, 8, 9, restricting condomless sex to peak fertility days, fertility screening to rule out subfertility, vaginal self‐insemination when the woman is living with HIV, and/or the use of fertility technologies, such as sperm washing with intrauterine insemination when the male partner is living with HIV or when fertility is compromised 10, 11, 12.

Many of the HIV prevention options used for safer conception are available through existing healthcare programmes, such as ART, VMMC and growing availability of PrEP. One barrier to fully minimizing HIV transmission risk during peri‐conception is knowledge about safer conception options among individuals and providers and the initiation of discussion about these methods (individually and as a package) between providers and patients 13, 14. Tools have been developed to empower providers to initiate discussions with couples about pregnancy plans 15 and guidance from the World Health Organization promotes safer conception counselling 16. Yet prevailing community perspectives and assumptions about sexual and perinatal HIV transmission by people living with HIV prevent many individuals and couples from actively seeking safer conception counselling or services 14, 17.

While many safer conception options are available and many have been proven effective against HIV transmission in general, more data are needed to understand when couples prefer certain methods and the feasibility of delivering a multi‐strategy intervention. Thus, the objective of this pilot study was to determine uptake, use and effectiveness of a comprehensive safer conception intervention among HIV‐serodiscordant couples with immediate fertility desires.

## 2 Methods

### 2.1 Study population

The Safer Conception Intervention for Partners (SCIP) pilot study was conducted in Thika, Kenya at the Kenya Medical Research Institute Partners in Health and Research Development (KEMRI‐PHRD) clinical research site from March 2016 to April 2018. The site has long‐established experience working with HIV‐serodiscordant couples in trials of PrEP and ART 5, 18, 19. Recruitment methods included partnering with existing HIV testing and counselling (HTC) centres and ART clinics, distribution of educational materials to couples on the benefits of couples‐based HTC, and talking with support groups for HIV‐serodiscordant couples. HIV‐serodiscordant couples were eligible for the study if both members were ≥18 years (and women ≤49 years), they expressed a desire for a pregnancy within the next three years, were sexually active with vaginal intercourse occurring at least six times in the past three months, were willing to enter the study as a couple, and intended to remain a couple for the study duration. Couples were excluded if there was any indication of subfertility or infertility (recent history of >12 months attempting pregnancy without success), if the woman was pregnant or breastfeeding or if she had used injectable contraception in the past three months, if the member living with HIV was enrolled in an HIV treatment study, or if the HIV‐negative member had abnormal renal function or active Hepatitis B infection. Finally, eligibility criteria also required that couples own a mobile phone that operated using a telecom network supported by the study short message service (SMS) platform, know how to receive and send SMS, and have access to electricity to charge the phone. Early in study recruitment, we used a validated pregnancy likelihood score to exclude couples unlikely to become pregnant (with a score <7) 20; this eligibility criteria was removed when we determined that the score was excluding couples with strong fertility desires meeting all other criteria.

### 2.2 Study design and procedures

This was an open‐label pilot study of a comprehensive safer conception intervention provided to all enrolled couples. Counsellor and clinician recommendations about specific safer conception strategies were tailored to each couple based on their preferences and ability to use each strategy. Couples attended monthly visits at the study clinic prior to pregnancy and quarterly visits during pregnancy. Couples were followed for 12 months or until the end of pregnancy, whichever was longer. During all visits, couples received counselling about HIV prevention 21 as well as information about how to track women's menstrual cycles and identify peak fertility days and how to conduct vaginal self‐insemination. Prior to pregnancy, women were provided with ovulation prediction kits (Clearblue) and counselling about how to use them. Couples that did not become pregnant after six months of pregnancy attempts were referred to fertility centres and remained in the study for at least six months longer.

At each visit, women were provided with β‐hCG urine pregnancy testing. HIV‐negative partners were provided with rapid HIV testing, counselled about PrEP, provided PrEP if desired, and counselled about daily adherence to PrEP. Participants dispensed PrEP were given a medication event monitoring (MEMS) cap for their pill bottle, which records a date‐time stamp every time the bottle is closed. Participants living with HIV were provided with counselling about the benefits of ART and ART adherence; CD4 count and HIV RNA were quantified every three months prior to viral suppression (defined as HIV RNA < 1000 copies/mL) and every six months thereafter. During monthly pre‐pregnancy visits and as clinically indicated during pregnancy, all participants were provided with diagnostic testing for *Neisseria gonorrhoeae, Chlamydia trachomatis and Trichomonas vaginalis* (Hologic Aptima Gen‐probe) and treated per national guidelines for active infections. Referrals were provided to male partners desiring VMMC and to men and women for fertility care when sperm washing or another assisted reproductive service was desired. Safety was monitored through reports of serious adverse events (SAE) by all participants and adverse events related to PrEP use for the HIV‐negative partners using PrEP. In addition, social harms were tracked carefully by the study team and the on‐site psychologist was engaged to provide services for mental health and gender‐based violence when needed.

The intervention included multiple mobile Health (“mHealth”) enhancements including: (1) a 6‐item daily pre‐pregnancy SMS survey completed by women to capture daily fertility indicators and sexual behaviour, (2) weekly SMS messages to men and women reminding them of peak fertility days during peri‐conception periods, and (3) a clinic‐based tablet‐application that collated fertility tracking, PrEP use, and viral load data and was used by providers to enhance safer conception counselling. Data describing the use of these strategies are presented elsewhere 22.

### 2.3 Statistical methods

Pregnancy incidence was calculated as the number of pregnancies divided by the total time women spent in the study prior to becoming pregnant since all women were considered to be fertile at enrolment. Women lost‐to‐follow up contributed time through their last attended study visit. Cumulative probability curves were estimated based on the time to first pregnancy and we used a Cox proportional hazards model to determine whether HIV status was associated with pregnancy incidence with adjustment for confounding factors.

We used methods for developing a counterfactual comparison in order to derive the expected number of incident infections in our cohort 18. Using data from the placebo arm of the Partners PrEP Study, a placebo‐controlled randomized trial of PrEP efficacy conducted from 2008 to 2011 among HIV‐serodiscordant couples, we simulated a comparable “non‐intervention” cohort, frequency matched to the SCIP study population by pregnancy likelihood score. Follow‐up time from participants in the Partners PrEP Study was censored to reflect the average follow‐up time among SCIP participants. The mean number of infections expected in the counterfactual population was averaged over 10,000 bootstrap samples and a 95% confidence interval was defined by the 2.5th and 97.5th percentiles. We descriptively compared pooled baseline information (median age, percentage of couples with a female HIV‐negative partner, median viral load and CD4 count of the partner living with HIV, HIV risk score 23, and proportion of follow‐up time pregnant in the study) from the complete set of counterfactual populations to the SCIP population to ensure similar characteristics of the groups. Once our counterfactual simulations were complete, we compared the estimated number of incident HIV infections in the counterfactual population (“expected” infections) with those actually observed in SCIP by computing incidence rate ratios. The 95% confidence interval for the incidence rate ratio was estimated using a Poisson distribution, and the p‐value was estimated by assessing the frequency of a comparable number of infections within the bootstrap sample. Additional bootstrap distributions were constructed with restriction to the age and gender of the HIV‐negative partner to create stratified estimates by age group and gender. In addition, we performed a separate set of calculations restricting the counterfactual dataset to include only couples from the Thika, Kenya site within the Partners PrEP Study. All analyses were conducted using SAS version 9.4 (SAS Institute, Cary, NC, USA) and Tableau 10.3 (Tableau Software, Seattle, WA, USA) was used to generate a treemap showing combinations of safer conception strategies.

### 2.4 Ethics

The study protocol was reviewed and approved by the Scientific Ethics Review Unit at KEMRI, the Human Subjects Division at the University of Washington, the Kenya Pharmacy and Poisons Board, and registered on clinicaltrials.gov (#NCT03030768). All participants separately provided written informed consent.

## 3 Results

### 3.1 Participant characteristics

**Figure 1:**
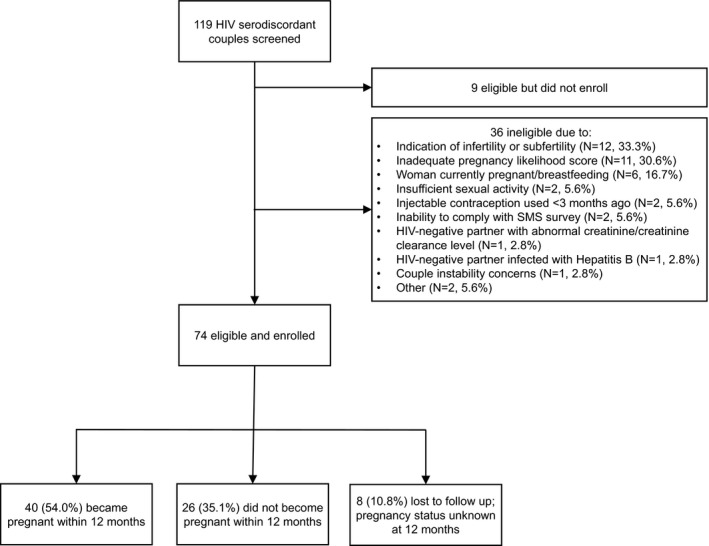
Enrolment and follow‐up of participants

Of the 119 couples screened, 83 were eligible and 74 enrolled (Figure 1). The primary reasons for screening out included an indication of infertility or subfertility (33.3%), a low pregnancy likelihood score (30.6%) and the woman being pregnant or breastfeeding (16.7%).

Among the 74 enrolled couples, 40 (54%) were female HIV‐negative/male HIV‐positive couples (Table 1). Women were a median age of 29.8 years (interquartile range (IQR) 27.0 to 35.1) and men were a median age of 35.3 years (IQR 29.9 to 40.1). Approximately one‐third (31.1%) of couples had children together and 87.2% wanted a child within the next year. One‐quarter (25.7%) reported trying to become pregnant recently and there was an overall median time of 0.5 years (IQR 0.1 to 0.8) that couples had spent attempting pregnancy during the past year. Couples reported a median of 8 to 12 sex acts together in the month prior to enrolment and 2.7% of participants reported sex with additional partner(s). Treatable STI were diagnosed infrequently: eight people were infected with *C. trachomatis*, two with *N. gonorrhoeae* and three with *T. vaginalis*. Prior to enrolment, 91.2% of women living with HIV had initiated ART (median ART use = 1.4 years, IQR 0.5 to 3.8) and 73.5% of women were virally suppressed. Among men living with HIV, 95.0% had initiated ART (median ART use = 2.8 years, IQR 0.4 to 6.3) and 82.5% were virally suppressed.

**Table 1:** Participant characteristics (Median, IQR or N, %)

	All participants (N = 148)	Couples with HIV‐negative women (N = 40 couples)		Couples with HIV‐positive women (N = 34 couples)	
		HIV‐negative women	HIV‐positive men	HIV‐negative men	HIV‐positive women
Demographic characteristics					
Age, years	32.8 (28.0 to 38.3)	31.0 (28.3 to 38.1)	37.1 (33.4 to 43.6)	34.6 (26.8 to 38.6)	28.9 (24.4 to 32.0)
Any income reported	131.0 (88.5%)	32.0 (80.0%)	40.0 (100.0%)	33.0 (97.1%)	26.0 (76.5%)
Education, years	10.0 (8.0 to 12.0)	10.0 (8.0 to 12.0)	10.0 (8.0 to 12.0)	10.0 (8.0 to 12.0)	10.0 (8.0 to 12.0)
Couple characteristics^a^					
Married	146.0 (98.7%)	39.0 (97.5%)	–	33.0 (97.1%)	–
Partnership duration, years	3.2 (1.8 to 8.8)	5.0 (2.0 to 9.8)	–	3.0 (1.3 to 7.1)	–
# children with study partner	0.0 (0.0 to 1.0)	0.0 (0.0 to 1.0)	–	0.0 (0.0 to 0.0)	–
# more children desired	2.0 (1.0 to 2.0)	2.0 (1.0 to 2.0)	2.0 (1.0 to 2.0)	2.0 (1.0 to 3.0)	2.0 (1.0 to 2.0)
Desired timing of next child					
Next year	129 (87.2%)	33 (82.5%)	34 (85.0%)	32 (94.1%)	30 (88.2%)
1 to 3 years	19 (12.8%)	7 (17.5%)	6 (15.0%)	2 (5.9%)	4 (11.8%)
Discussed fertility desires with partner prior to enrolment	142 (96.0%)	39 (97.5%)	39 (97.5%)	31 (91.2%)	33 (97.1%)
Recently tried to become pregnant prior to enrolment	38 (25.7%)	9 (22.5%)			10 (29.4%)
Time trying to conceive prior to enrolment, years	0.5 (0.1 to 0.8)	0.4 (0.2 to 0.6)			0.5 (0.1 to 0.8)
Time known HIV serodiscordant, years	1.0 (0.2 to 4.0)	1.8 (0.3 to 4.7)		0.8 (0.1 to 3.0)	
Sexual behaviour and clinical characteristics in month prior to enrolment					
# sex acts with study partner^a^	10.0 (5.0 to 12.0)	8.0 (4.5 to 12.0)	–	12.0 (8.0 to 16.0)	–
Any condomless sex with study partner^a^	56.0 (37.8%)	12.0 (30.0%)	–	15.0 (44.1%)	–
Any sex with additional partner(s)	4.0 (2.7%)	0.0 (0.0%)	2.0 (5.0%)	2.0 (5.9%)	0.0 (0.0%)
Circumcised (men only)	70.0 (94.6%)	–	38.0 (95.0%)	32.0 (94.1%)	–
Infected with *C. trachomatis*	8 (5.4%)	2 (5.0%)	0 (0.0%)	5 (14.7%)	1 (2.9%)
Infected with *N. gonorrhoeae*	2 (1.4%)	2 (5.0%)	0 (0.0%)	0 (0.0%)	0 (0.0%)
Infected with *T. vaginalis*	3 (2.0%)	0 (0.0%)	0 (0.0%)	1 (2.9%)	2 (5.9%)
HIV–1 characteristics					
CD4 count (cells/μl)	568 (389 to 735)	–	475 (384 to 660)	–	649 (441 to 830)
Self–report > 3 mo ART use	53.0 (71.6%)	–	30.0 (75.0%)	–	23.0 (67.6%)
Plasma HIV RNA (log_10_ copies/mL)	1.6 (1.6 to 1.9)	–	1.6 (1.6 to 1.9)	–	1.6 (1.6 to 2.7)
Virally suppressed at enrolment^b^	58.0 (78.4%)	–	33 (82.5%)	–	25 (73.5%)
Initiated PrEP	74.0 (100.0%)	40.0 (100.0%)	–	34.0 (100.0%)	–

ART, antiretroviral therapy; PrEP, pre–exposure prophylaxis.

^a^Data shown in the HIV–negative group apply to the couples (if there were reporting discrepancies between the partners’ information, the data from the HIV–negative member of the couple was used); ^b^participants were considered to be virally suppressed if they had plasma HIV RNA < 400 copies/mL.

### 3.2 Retention

Of the 148 individuals (from 74 couples) enrolled, 96.6% had at least one study follow‐up visit and 77% were active in the study through the end of their study‐defined follow‐up period. Women from eight couples were lost to follow‐up before pregnancy occurred. The study accrued a total of 146.4 (72.6 from men and 73.8 from women) person‐years of follow‐up. Three couples exited the study early due to a change in their fertility intentions and no longer desiring pregnancy.

### 3.3 Pregnancy incidence

**Figure 2:**
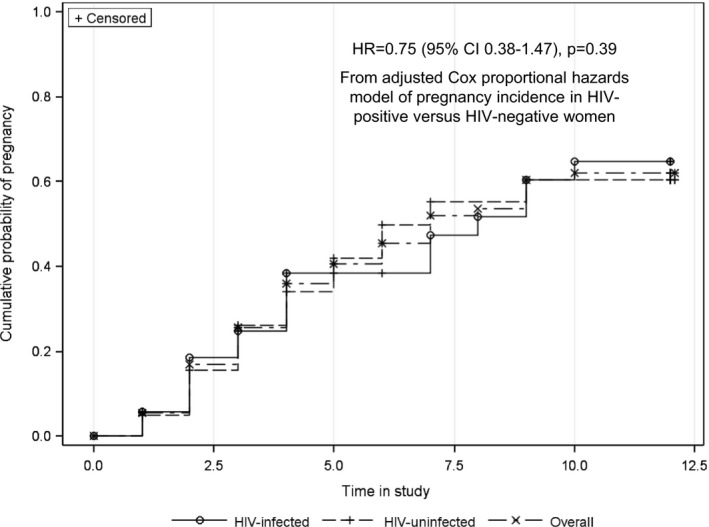
Cumulative probability of HIV infection, overall and by HIV status

Forty couples (54.0%) became pregnant, including seven who had an early miscarriage and became pregnant a second time during the study. The cumulative probability of first pregnancy was 45.3% at 6 months (38.5% for women living with HIV and 49.9% for HIV‐negative women) and 61.9% at 12 months (64.8% for women living with HIV and 60.4% for HIV‐negative women). The average time to pregnancy was 6.1 months (median = 7 months, IQR 5 to 10). There was no statistical difference in the time to pregnancy between women living with HIV and HIV‐negative women (Figure 2, *p* = 0.39). Pregnancy outcomes included 26 (55.3%) live births (24 term and two pre‐term), 15 (31.9%) pregnancy losses (14 at <13 weeks and 1 at 13 weeks gestation) and six pregnancies were ongoing at the conclusion of the study. There were no ectopic pregnancies and all babies were HIV negative at birth. Among 34 couples who did not become pregnant, 17 (50%) had an indication of not sustaining their pregnancy attempts (three exited the study early due to a change in fertility desires, six reported a break‐up in their relationship before their follow‐up time concluded and eight were lost to follow‐up).

### 3.4 Uptake and use of safer conception strategies

Overall, 89.2% of couples maintained their relationships throughout the study and the uptake of safer conception strategies was high (Table 2).

**Table 2:** Couple characteristics, sexual behaviour and HIV risk considerations during follow‐up^a^

	Among couples who became pregnant		Among couples who did not become pregnant
	During the month prior to pregnancy (N = 40 couples; 47 study visits)	During the three months prior to pregnancy (N = 36 couples; 101 study visits)^b^	During the entire follow‐up period (N = 34 couples; 424 study visits)^c^
Characteristics			
Proportion of couples who were in a relationship with their study partner during the period	40/40 (100.0%)	35/36 (97.2%)	27/34 (79.4%)
Frequency of visits when women reported trying to get pregnant	34/47 (72.3%)	70/101 (69.3%)	175/424 (41.3%)
Sexual behaviour			
Median number of monthly sex acts during period, reported in SMS messages	10.0 (7.0 to 12.0)	10.0 (6.0 to 18.0)	7.0 (1.0 to 12.0)
Median number of monthly condomless sex acts, reported in SMS messages	3.5 (1.0 to 7.0)	4.0 (1.0 to 9.0)	3.0 (1.0 to 6.0)
ART use by the partner living with HIV			
Proportion of visits with ART use	47/47 (100.0%)	101/101 (100.0%)	421/424 (99.3%)
Median number of months with ART use	1.0 (1.0 to 1.0)	3.0 (3.0 to 3.0)	12.0 (12.0 to 12.0)
Proportion of visits virally suppressed	38/47 (80.9%)	75/101 (74.3%)	274/424 (64.6%)
Median number of months with viral suppression during the period	1.0 (1.0 to 1.0)	3.0 (2.0 to 3.0)	7.0 (4.0 to 12.0)
PrEP use by the HIV‐negative partner			
Number of visits when PrEP was dispensed	43/47 (91.5%)	84/101 (83.2%)	271/424 (63.4%)
Proportion of pregnancies^d^ with PrEP dispensed during all visits in the period	43/47 (91.5%)	24/36 (66.7%)	11/34 (32.4%)
Median number of months PrEP used during the period^c^	1.0 (1.0 to 1.0)	3.0 (2.0 to 3.0)	9.0 (4.0 to 11.0)
MEMS adherence >80% during entire period	35/43 (81.4%)	68/84 (81.0%)	195/271 (72.0%)
Proportion of pregnancies^d^ with MEMs adherence >80% during all visits in the period while on PrEP	35/43 (81.4%)	26/34 (76.5%)	23/32 (71.9%)
Median number of months with MEMS‐indicated adherence > 80%	1.0 (1.0 to 1.0)	3.0 (2.0 to 3.0)	6.0 (2.0 to 10.5)
Median number of monthly PrEP pill bottle openings	28 (24 to 30)	28 (24 to 29)	27 (21 to 29)
Female had STI diagnosis	0/47 (0.0%)	0/101 (0.0%)	0/424 (0.0%)
Median number of SMS fertility tracking surveys completed per month	27.0 (23.0 to 29.0)	27.0 (23.0 to 29.0)	25.0 (14.0 to 28.0)
Male partner was circumcised	47/47 (100.0%)	101/101 (100.0%)	418/424 (98.6%)
Couple reported ever using vaginal self‐insemination	0/47 (0.0%)	0/101 (0.0%)	3/424 (0.7%)
Couple reported ever using trying sperm washing	0/47 (0.0%)	2/101 (2.0%)	0/424 (0.0%)
Couple reported ever using artificial insemination, IUI or ICSI	0/47 (0.0%)	0/101 (0.0%)	0/424 (0.0%)

Sexual behaviour data are based on the reports from the woman. ART, antiretroviral therapy; SMS, short message service; STI, sexually transmitted infections.

^a^Data are shown as N(%) or median (IQR); ^b^excludes four pregnancies that occurred before the couple had three months of follow‐up time; ^c^includes visits prior to study exit (which occurred at 12 months for 31 couples and <12 months for three couples who exited the study early) and follow‐up visits after the last attended visit for eight couples who were lost to follow‐up and had unknown pregnancy status at 12 months; ^d^for couples who did not become pregnant, the number shown is the proportion of people.

### 3.5 ART use

All participants living with HIV reported ART use by their one‐month follow‐up visit. Prior to pregnancy, 100% of partners living with HIV used ART, 80.9% were virally suppressed during the month prior to pregnancy, and viral suppression was present during 74.3% of visits in the 3‐month period prior to pregnancy. At 64.6% of visits by partners living with HIV in a couple that did not become pregnant, the person was virally suppressed.

### 3.6 PrEP use

One‐hundred percent (100%) of HIV‐negative partners initiated PrEP. In the month prior to pregnancy, PrEP was dispensed at 91.5% of visits and 81.4% of people took >80% of expected doses. During the three months prior to pregnancy, PrEP was dispensed at 83.2% of visits and 81.0% of people took >80% of expected doses. PrEP dispensing and adherence was slightly less among people who were in a couple that did not become pregnant (63.4% dispensed and 72.0% with > 80% expected doses taken).

### 3.7 Fertility tracking and sexual behaviour

Seventy percent (70%) of daily surveys to track fertility indicators were fully answered. During the month prior to pregnancy, women reported a median of 10.0 (IQR 7.0 to 12.0) total sex acts, of which a median of 3.5 (IQR 1.0 to 7.0) were without a condom, indicating attention to fertility tracking and potentially limiting condomless sex to days with predicted peak fertility. During the three months prior to pregnancy, women reported a median of 10.0 (IQR 6.0 to 18.0) total sex acts per month, including 4.0 (IQR 1.0 to 9.0) without a condom. Women who did not become pregnant reported a median of 7.0 (1.0 to 12.0) total sex acts per month, including 3.0 (IQR 1.0 to 6.0) without a condom.

### 3.8 Additional safer conception strategies

All but four men were circumcised at enrolment and three of these became circumcised <3 months after enrolment. One woman reported using vaginal self‐insemination during a study visit six months prior to her first positive pregnancy test. Two women reported using sperm washing, both during study visits within three months of their first positive pregnancy test. There were no reports of artificial insemination attempts or use of intrauterine insemination or intracytoplasmic sperm injection.

### 3.9 Strategy combinations

**Figure 3:**
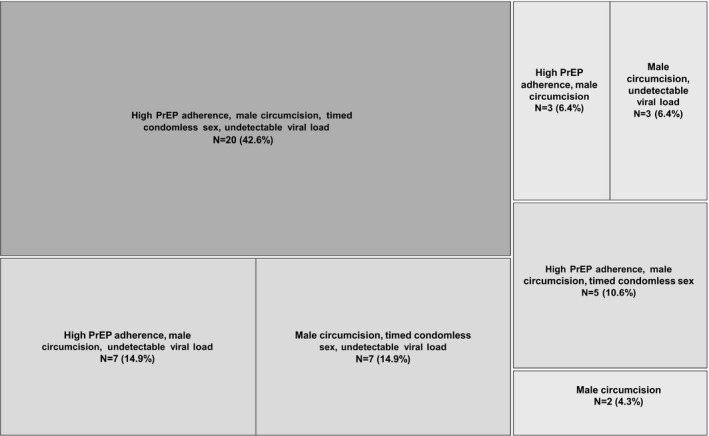
Use of safer conception strategies in the month prior to pregnancy (N = 47 pregnancies) High PrEP adherence is defined as 80% of expected doses based on MEMS data; timed condomless sex defined as having fewer condomless sex acts than the total reported through daily SMS surveys.

Most (95.7%) of pregnancies were covered by ≥2 strategies (Figure 3). During the month prior to pregnancy, all men were circumcised and many couples also had high adherence to PrEP, ART and timed condomless sex: 20 (42.6%) pregnancies were protected by the use of all four of these strategies. For the remainder, in addition to male circumcision, seven pregnancies (14.9%) were also protected by high adherence to PrEP and ART, 5 (10.6%) were protected by PrEP and timed condomless sex (all of these couples were using ART but without viral suppression), 7 (14.9%) were protected by viral suppression and timed condomless sex (four of these were using PrEP but without high adherence), 3 (6.4%) had PrEP and 3 (6.4%) had viral suppression (one was using PrEP but without high adherence). Only 2 (4.3%) couples did not add a protective measure beyond male circumcision.

### 3.10 SAE and social harms

Of the 39 total SAE, five were grade 4 related to incomplete abortion, miscarriage, hypertension and attempted suicide. The remainder were grade 3, of which the majority (21/34, 61.8%) were hospitalizations due to pregnancy delivery. No SAE were related to PrEP or ART. Social harm events were reported 21 times, including 14 incidents reported by participants living with HIV (thirteen women and one man) and seven incidents among HIV‐negative participants (six women and one man). Of the non‐mutually exclusive types of social harm, 90.5% of reports included an element of verbal abuse, 57.1% included a report of physical abuse and 47.6% included a report of economic abuse. Two‐thirds of these social harms (66.7%) resulted in relationship discontinuation and one event resulted in a pregnancy loss. Two of the social harm events resulted in hospitalization. One social harm was related to study SMS messages when a male partner mistook the messages as being from a former partner.

### 3.11 Intervention effectiveness against HIV transmission

We observed no HIV seroconversions (95% CI 0.0 to 6.2, Table 3). Using a comparable counterfactual cohort derived from the placebo arm of the Partners PrEP Study, we estimated that we would have expected to observe 1.9 to 3.0 incident HIV infections, given the HIV risk characteristics of our sample. Using gender matching and restricting couples in our counterfactual cohort to those from the Thika site, we estimated an incidence rate ratio (IRR) of 0.00 (*p* = 0.04) demonstrating a statistically significant 100% reduction in HIV incidence with the use of a comprehensive safer conception package. Age‐matching produced a similar statistically significant estimate (IRR = 0.0, *p* < 0.001).

**Table 3:** Estimated effectiveness of a comprehensive safer conception package to prevent HIV transmission based on a historical cohort

	Expectation from the Partners PrEP study		Observed from the SCIP study		Incidence rate ratio (95% CI)	*p*‐value
	N incident infections/N years of follow‐up^a^	Incidence (95% CI)^b^	N incident infections/N years of follow‐up^a^	Incidence (95% CI)^b^		
All sites in the Partners PrEP Study						
Overall incidence^c^	2.1/64.8	3.23 (0.0, 8.0)	0.0/61.6	0.00 (0.0, 6.0)	0.00 (Undefined)	0.12
By gender^d^	3.0/64.6	4.67 (0.0, 10.9)	0.0/61.6	0.00 (0.0, 6.0)	0.00 (Undefined)	0.04
By age^e^	1.9/65.5	2.90 (0.0, 7.8)	0.0/61.6	0.00 (0.0, 6.0)	0.00 (Undefined)	0.15
Restricting to the Thika, Kenya site from the Partners PrEP Study						
Overall incidence^c^	2.9/65.1	4.39 (0.0, 9.8)	0.0/61.6	0.00 (0.0, 6.0)	0.00 (Undefined)	0.05
By gender^d^	2.9/65.4	4.36 (0.0, 9.6)	0.0/61.6	0.00 (0.0, 6.0)	0.00 (Undefined)	0.04
By age^e^	2.6/65.0	3.99 (1.5, 8.0)	0.0/61.6	0.00 (0.0, 6.0)	0.00 (Undefined)	<0.001

^a^The number of expected seroconversions and person‐years do not sum precisely to the overall totals because each subgroup estimate is drawn from a separate bootstrapped counterfactual cohort model; ^b^Per 100 person‐years. The incidence is calculated with a modified intention‐to‐treat analysis approach, where Partners PrEP participants who were found to have prevalent HIV infection at enrolment were excluded from the sample; ^c^In this analysis, the counterfactual population was sampled to match the distribution of pregnancy risk score in the SCIP sample; ^d^In this analysis, the counterfactual population was sampled to match the distribution of pregnancy risk score and gender of the HIV‐negative partner in the SCIP sample; ^e^In this analysis, the counterfactual population was sampled to match the distribution of pregnancy risk score and age of the HIV‐negative partner in the SCIP sample.

## 4 Discussion

In this pilot study for HIV‐serodiscordant couples in Kenya desiring pregnancy, we found high (95.7%) uptake of safer conception strategies used in combination, especially combinations of ART, PrEP, male circumcision and condomless sex timed to peak fertility. These combined prevention measures resulted in no HIV sexual transmission, which was a statistically significant reduction in the estimated HIV risk we were predicted to observe. Providing couples with multiple preventions options was important as not one intervention was used 100% of the time leading up to pregnancy. Importantly, there was not 100% HIV viral suppression leading up to all pregnancies and during the months of pregnancy attempts. Thus, HIV prevention strategies complementary to ART filled an important prevention gap. While reports were rare, two women reported using sperm washing and one reported self‐insemination, although these reports were >1 month preceding pregnancy and unlikely to have resulted in pregnancy.

Primary pieces of safer conception implementation that need focused efforts now are the empowerment of healthcare providers to recognize need and initiate effective conversation and the empowerment of individuals and communities to seek safer conception services. Safer conception programming also needs to be broad enough to include individuals who are unable or unwilling to engage their partner in HIV status disclosure and HIV prevention services. For many individuals and couples, the gateway to practicing safer conception is communication between themselves and a healthcare provider about pregnancy goals. In our cohort, study staff initiated these conversations with couples during the screening process and it was the first experience for many of the couples to discuss their ideal family size and timing of the next pregnancy in detail with a healthcare provider as well as with each other. It is also important to recognize and prepare providers that focused attention on pregnancy plans can have unintended negative consequences within a couple, such as partnership dissolution and social harm that can warrant referral to psychological counselling. Our findings about social harms warrant further investigation within ongoing safer conception programmes and research contexts, including with qualitative research permitting documentation of complete experiences.

In sub‐Saharan Africa, ART and VMMC are widely available to the general population and PrEP delivery is being scaled up quickly in many different settings. In Kenya, for example, PrEP is available through ART, antenatal, and family planning clinics, comprehensive health services for men who have sex with men and female sex workers and implementation is growing in other countries, especially for key populations and HIV‐serodiscordant couples 24. We were able to enhance our intervention with frequent HIV viral load monitoring, mHealth tools and MEMS caps to track PrEP adherence. Less resource intense programmes can prioritize sustained ART use with high adherence (with or without viral load monitoring), PrEP to fill prevention gaps when HIV viraemia is unsuppressed and when desired, and tracking fertility indicators via paper calendar to enable timing condomless sex, reflecting lower cost and less time consuming delivery of the safer conception strategies most commonly used by the couples in our pilot.

Many recent safer conception studies and public programmes have shown good uptake of safer conception strategies, including ART and PrEP 25, 26, 27, 28, 29. To the best of our knowledge, ours is the first study to estimate the effectiveness of a safer conception intervention on HIV transmission risk. The strength of our counterfactual simulation is that we were able to identify couples from the same geographic region with age distribution and pregnancy incidence similar to our safer conception cohort. However, the comparison population is limited because ART was much more available in 2016 to 2018 than it was in 2008 to 2011, resulting in a large proportion of our cohort being virally suppressed at baseline. Nonetheless, we observed no HIV transmissions among our study couples who had a median baseline HIV risk score of five which corresponds to an expected HIV incidence of 3% per year 23. Another limitation of our study is that we enrolled a cohort with median age of 30, likely related to eligibility criteria prioritizing stable couples who were truly intending to conceive. Thus, our results may not be fully generalizable to younger, newly formed couples. A limitation of our current analysis is that we have used a fairly crude definition for timed condomless sex. Further evaluation of couple's ability to use condoms except during days with peak fertility is necessary.

## 5 Conclusions

In conclusion, this pilot of a comprehensive safer conception intervention effectively eliminated sexual HIV transmission and demonstrated high uptake of combination safer conception strategies, with preferences for strategies that are already available in public settings in sub‐Saharan Africa. These findings reinforce international calls to scale‐up programmatic delivery of safer conception services as a package intervention that promotes counselling on fertility desires and emphasizes the utility of ART, PrEP and timed condomless sex for safer conception 30. Public health priorities include advancing the integration of safer conception counselling and services into existing healthcare programmes, engaging communities to promote acceptability of pregnancy among people affected by HIV, and expanding the number of service providers that are empowered to encourage discussion of pregnancy desires with HIV‐affected individuals and couples.

## Competing interests

The authors report no conflicts of interest.

## Authors’ contributions

RH, KN, NM and JMB designed the study. JV conducted all statistical analyses. All authors approved the final version of the manuscript. RH contributed to funding, study design, results interpretation and manuscript first draft. KN contributed to study design, results interpretation and edited manuscript. JV contributed to statistical analysis, results interpretation and edited manuscript. CK, JQ, LO, NT, JN, RM, GM, SM, HH and BC contributed to data collection and edited manuscript. CC contributed to results interpretation and edited manuscript. JMB involved in study design, results interpretation and edited manuscript. NM contributed to study design, results interpretation and edited manuscript.
